# A somatic mutation in *PIK3CD* unravels a novel candidate gene for lymphatic malformation

**DOI:** 10.1186/s13023-021-01782-9

**Published:** 2021-05-08

**Authors:** Shengcai Wang, Wei Wang, Xuexi Zhang, Jingang Gui, Jie Zhang, Yongli Guo, Yuanhu Liu, Lin Han, Qiaoyin Liu, Yanzhen Li, Nian Sun, Zhiyong Liu, Jiangnan Du, Jun Tai, Xin Ni

**Affiliations:** 1 Department of Otolaryngology-Head and Neck Surgery, Beijing Children’s Hospital, Capital Medical University, National Center for Children’s Health, Beijing, 100045 China; 2 Laboratory of Tumor Immunology, Beijing Pediatric Research Institute, Beijing Children’s Hospital, Capital Medical University, National Center for Children’s Health, Beijing, 100045 China; 3 Beijing Key Laboratory for Pediatric Diseases of Otolaryngology, Head and Neck Surgery, Beijing Pediatric Research Institute, Beijing Children’s Hospital, Capital Medical University, National Center for Children’s Health, Beijing, 100045 China; 4 Running-Gene Inc., Health Valley 602, Beijing, China

**Keywords:** Lymphatic malformations, Whole-exome sequencing, *PIK3CD*, Novel mutations, MTOR pathway

## Abstract

**Background:**

Lymphatic malformations (LMs) are benign congenital malformations that stem from the abnormal development of the lymphatic vessels during early embryogenesis. Somatic *PIK3CA* gene mutations are conventional cause leading to LMs. Both macrocystic and microcystic LMs arise due to lymphatic endothelial cell-autonomous defects, depending on the time in development at which *PIK3CA* gene mutation occurs. Recent study finds a PIK3CA mutation in 79% of LMs. However, discovering new genetic events in this disease is crucial to identify the molecular mechanism of the pathogenesis and further develop new targeted therapies.

**Results:**

Here, we initially performed whole-exome sequencing in six children with LMs to find a new causal gene. Somatic mutations in *PIK3CA* (c.1633G > A [p. E545K] and *PIK3CD* (c.1997T > C [p.L666P]) were discovered in two different individuals. In vitro functional studies were conducted to demonstrate the pathogenicity of the novel mutation c.1997T > C in *PIK3CD*. We found that L666P promoted the cell proliferation and migration of human umbilical vein endothelial cells (HUVECs) and induced hyperactivation of the mTOR pathway. These findings indicate that the *PIK3CD* mutation affects downstream signalling in endothelial cells, which may impair normal lymphangiogenesis.

**Conclusions:**

This study reveals a novel candidate gene associated with the development of LMs, which is consistent with previous researches. These findings in our study may offer a novel gene target for developing therapies, which acts in tight interaction with the previously known PIK3CA.

**Supplementary Information:**

The online version contains supplementary material available at 10.1186/s13023-021-01782-9.

## Introduction

Lymphatic malformations (LMs) are congenital lesions caused by defects in the development of the lymphatic system and mainly observed in neonates or young children, with an incidence of 1 in 4000 to 1 in 2000 [1]. Lymphatic malformations (LM) are characterized by the overgrowth of lymphatic vessels during pre- and postnatal development [2]. LMs can cause adjacent structures compromise leading to airway obstruction even dyspnea, cosmetic deformity, swallowing impairment, infection or naturally diffuse, especially in head and neck region. Conservative observation and surgery are main treatments and the latter including surgical excision (e.g., partial or total excision), sclerotherapy, radiofrequency ablation, laser therapy. With the development of medical genetics of LMs, new therapies increasingly emerged such as oral medications (i.e., sildenafil, propranolol, and sirolimus), and vascularized lymph node transfer [3, 4]. A systematic review recruited 20 trials including 71 patients with oral sirolimus, and found that the sirolimus was effective for LMs [4]. However, patients with extensively infiltrating LMs, namely, intractable LMs (iLM), experienced high risks of recurrence and progression, routine treatment regimens are less effective for iLMs [5].

Recently, increasing evidence shows that the PI3K/AKT/mTOR pathway is involved in the pathogenesis of isolated LMs and syndromic disorders in which LM is a component feature [6–9]. For example, proteus syndrome patients who also have LMs carried a somatic mutation in *AKT1*, which encodes RAC-alpha serine-threonine protein kinase and plays a role in lymphangiogenesis [9, 10]. Moreover, somatic mutations that activate phosphatidylinositol 4,5-bisphosphate 3-kinase catalytic subunit alpha (PIK3CA) have been found in approximately 79% of LMs [8, 11]. Thus around 20% remain unexplained. Somatic *PIK3CA* mutation is not only identified in isolated LM, but also in CLOVES syndrome or Klippel–Trenaunay–Weber syndrome [9]. High activity of the PI3K-AKT- mTOR pathway was demonstrated by hyperphosphorylation of AKT-Ser473 in all LM-derived lymphatic endothelial cells (LECs) as compared to normal LECs while LM-derived fibroblasts did not possess such mutations [12]. Several *PIK3CA* somatic mutations have been shown to be oncogenic as well as function as the main pathogenic mechanisms of LMs and vascular malformations by promoting the hyperproliferation of endothelial cells [13]. The International Society for the Study of Vascular Anomalies (ISSVA) has also identified a *PIK3CA* mutation as a specific pathogenic cause for LMs. However, the molecular mechanism of LMs without *PIK3CA* mutations is still unclear, and other genetic alterations have not been found in the disease. Thus, the discovery of new genetic events in LMs is crucial to identify the molecular mechanism of the pathogenesis and further develop novel targeted therapies. In this study, a novel candidate mutation in *PIK3CD* was identified as an LM-associated mutation by whole-exome sequencing (WES) and was validated by in vitro functional studies.

## Results

### Diagnosis and characteristics of LMs

**Fig. 1:**
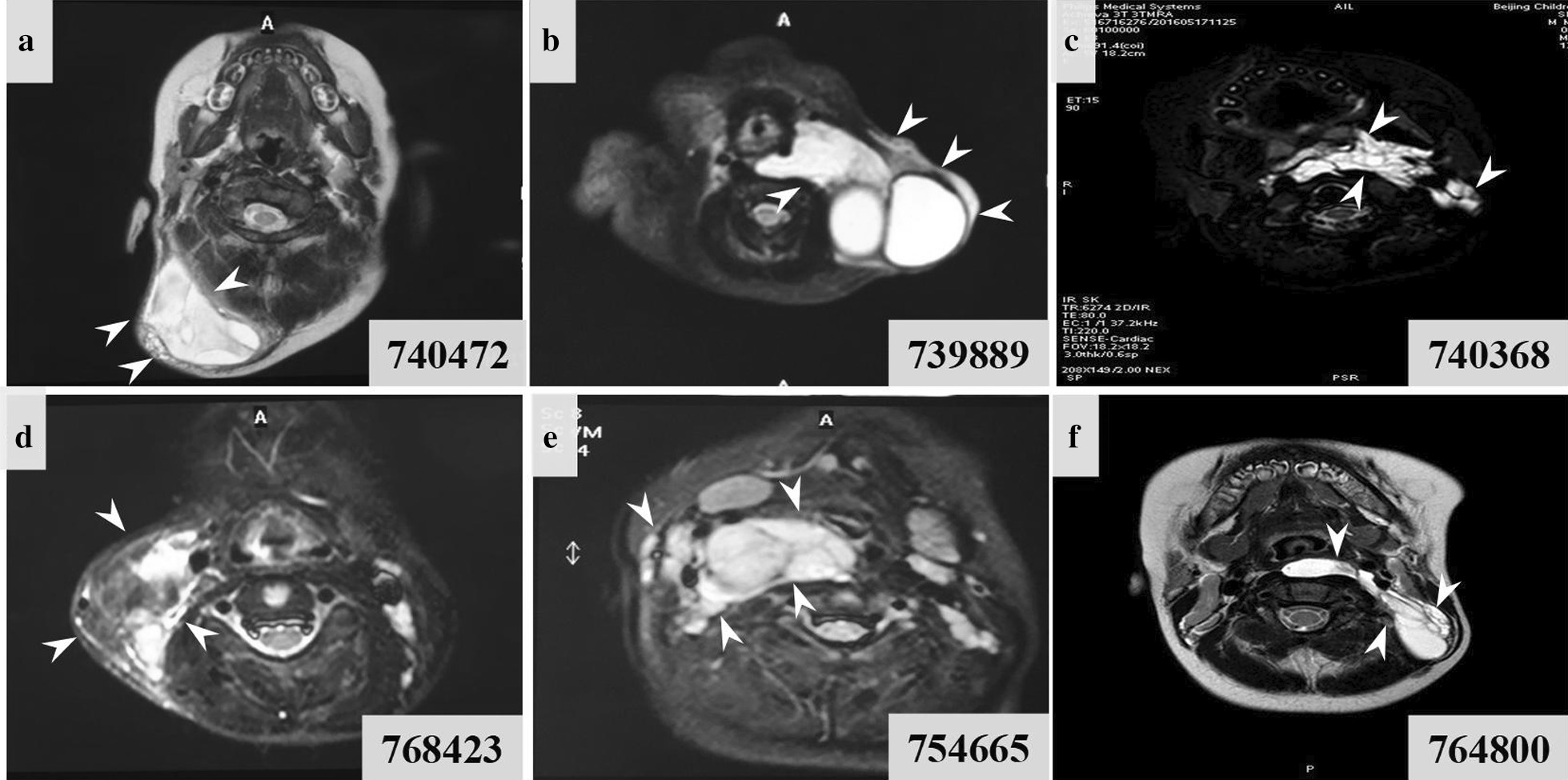
MRI image of each subtype of LMs. **a**, **b** MRI (T2 image in axial view) reveal macro-cystic lesions (more than 2 cm in diameter) in head and neck region. **c** MRI (T2 image in axial view) reveals micro-cystic lesion in parapharyngeal spaces, retropharyngeal space, and prevertebral space. **d** The axial postgadolinium contrast enhanced T1-weighted MRI LM with microcysts, with enhancing setae between the individual cysts. **e**, **f** MRI (T2 image in axial view) reveal macrocysts and microcysts

Preoperative diagnoses were based on the clinical and radiological findings (Table 1). The maximum diameter of every single cyst was measured by magnetic resonance imaging (MRI). Based on the size of the cyst, the LMs were morphologically classified as macrocystic LMs (the smallest cyst more than 2 cm in macrocystic malformations), microcystic LMs (the biggest cyst less than 2 cm in microcystic malformations) or mixed cystic LMs (containing both macrocystic and microcystic proportion of malformations). Axial T2 MRI demonstrated that most of the cervical LMs with high signal intensity comprised fluid component and septations, while heterogeneous areas of hypointensity usually suggested a haemorrhage. The extensive lesions caused airway obstruction, involving the trachea, blood vessels, parapharyngeal spaces, retropharyngeal space and prevertebral space (Fig. 1). Macrocystic LMs (ID: 740472, 739889) were composed of vessels with a single layer of flattened epithelial cells, including smooth muscle cells, with variable thickness. Microcystic LMs (ID: 740368, 768423) were irregularly shaped lesions resulting from the localized collection of abnormal and cystically dilated lymph vessels filled with lymph fluid. Mixed cystic LMs (ID: 754665, 764800) contained morphology representative of a mixture of micro- and macrocystic LMs (Additional file 1: Figure S1).

**Table 1:** Clinical features of children with lymphatic malformations

Patient ID	Age at surgery	Sex	Family history	Pathology	MRI report	Diagnose
740472	9 mo	Male	Normal	Lymphatic malformation with intrathecal haemorrhage	A 7.8*3.0*7.4 cm mass is seen in the right neck and occiput, long T1 and T2 signals	Macrocystic lymphatic malformation
739889	2 mo	Male	Normal	Lymphatic malformation with intrathecal haemorrhage	A 3.7*6.5*6.3 cm mass is seen in the left neck, long T1 and T2 signals, involving left parapharyngeal and retropharyngeal spaces and causing airway obstruction	Macrocystic lymphatic malformation
740368	25 mo	Male	Normal	Lymphatic malformation	Giant and long T1 and T2 signals in the left neck, involving bilateral parapharyngeal spaces, retropharyngeal space, prevertebral space and causing airway obstruction	Microcystic lymphatic malformation
768423	91 mo	Male	Normal	Lymphatic malformation with intrathecal haemorrhage	A 6*2*4 cm mass is seen in the right neck, short T1 and T2 signals	Microcystic lymphatic malformation
754665	32 mo	Female	Normal	Lymphatic malformation with intrathecal haemorrhage	A 4.8*1.8*4.0 cm mass is seen in the right neck, slightly short T1 and T2 signals	Mixed-cystic lymphatic malformation
764800	18 mo	Male	Normal	Lymphatic malformation	A 7.5*1.5*4.4 cm mass is seen in the left neck, long T1 and T2 signals, involving retropharyngeal space and causing airway obstruction	Mixed-cystic lymphatic malformation

### Mutations in PIK3CA and PIK3CD were identified in children with LMs

**Fig. 2:**
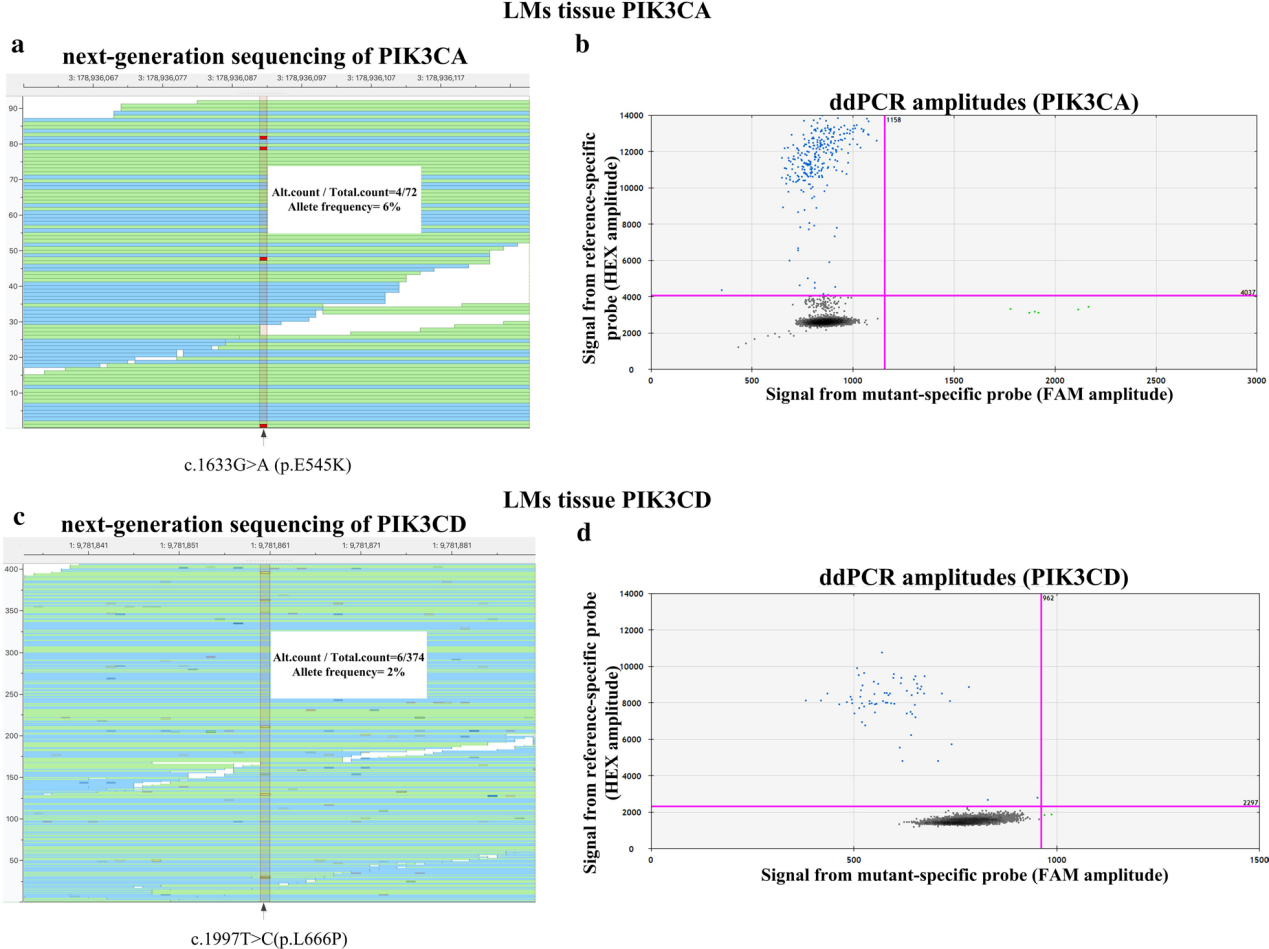
Results from WES and digital PCR verification. Binary alignment map of WES and scatter plot of digital PCR verification. Mutation c.1633G > A in the PI3KCA gene was identified by WES and verified by digital PCR (**a**, **b**). Mutation c.1997 T > C in the PI3KCD gene was identified by WES and verified by digital PCR (**c**, **d**). Panels **a** and **c** show read alignment of WES

**Fig. 3:**
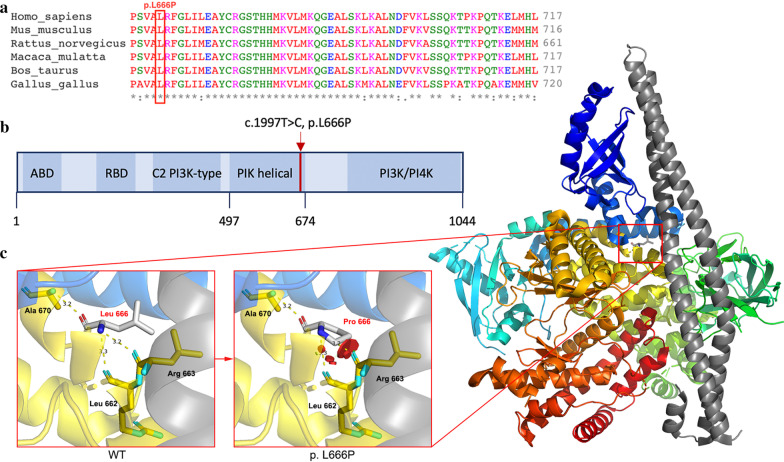
Structural analysis of PIK3CD. **a** Lysine 666 is highly conserved across multiple species. **b** PI3KCD encodes p110δ protein, which consists of an adaptor-binding domain (ABD), a Ras-binding domain (RBD), a C2 domain, an accessory domain (PIK helical) and a PI3K/PI4K catalytic domain. Mutation c.1997 T > C is located in the PIK helical domain. **c** Three-dimensional structure of p110δ shown in colour. After the alteration of L666P, repulsion will occur between Pro 666 and Arg 663 as well as Pro 666 and Leu 662, resulting in the instability of protein structure

Peripheral blood samples and lymphatic tissue specimens from 6 patients were collected for WES and digital polymerase chain reaction (PCR) verification. A total of 16 variants associated with mTOR pathway were identified in 6 genes, including *IRS1*, *MTOR*, *PIK3CA*, *PIK3CD*, *TSC1* and *TSC2*. For other candidate variants, no mutant droplet is detected (mTOR and TSC2) or ddPCR failed (TSC1 and IRS1, primers failed to distinguish wt and mut sequences). Finally, only two variants, c.1633G > A (p. E545K) in *PIK3CA* (NM_006218) and c.1997T > C (p.L666P) in *PIK3CD* (NM_005026), were identified in 2 patients (Table 2 and Fig. 2). The mutation frequency of *PIK3CA* was 6.06% (4/66) as detected by WES and 2.14% (6/281) by digital PCR, and that of *PIK3CD* was 1.73% (5/289) and 2.81% (2/71), respectively. We analysed the pathogenicity of the mutations mainly based on criteria from the Association for Molecular Pathology (AMP) Clinical Practice guidelines [14]. Mutation of c.1633G > A in *PIK3CA* has been reported as a disease-causing mutation associated with lymphatic disorders. The novel mutation c.1997T > C in *PIK3CD* has never been reported before in public databases for somatic mutations (COSMIC). Functional predictions by multiple software programs (MutationTaster, Provean and PolyPhen-2) demonstrated that these mutations are damaging and deleterious. The site of *PIK3CD* mutation, lysine 666, is highly conserved across many species and is located in the PIK helical domain (Fig. 3). Both mutations are associated with the PI3K/AKT/mTOR pathway; thus, we considered them variants with potential clinical significance. It should be noted that somatic activating mutations in the *PIK3CA* gene have been detected in LMs, but this is the first report of the *PIK3CD* mutation in this disease.

**Table 2:** Two variants were identified in children with LMs

Patient	Gene	RefSeq	Chromo-some	Nucleotide	Amino acid	Mutation Taster	SIFT (cutoff = 0.05)	Provean (cutoff = −2.5)	Polyphen-2	Reference/alteration (frequency)
740368	PIK3CA	NM_006218	chr3:178,936,091	c.G1633A	p.E545K	Disease causing (1.000)	Damaging (0.002)	Deleterious (-3.28)	Probably damaging (0.991)	WES: 62/4 (0.06) ddPCR: 275/6 (0.02)
739889	PIK3CD	NM_005026	chr1:9,781,860	c.T1997C	p.L666P	Disease causing (1.000)	Damaging (0.001)	Deleterious (-6.33)	Probably damaging (1.000)	WES: 284/5 (0.02) ddPCR: 69/2 (0.03)

### The PIK3CD mutation promoted cell proliferation and migration in human umbilical vein endothelial cells (HUVECs)

**Fig. 4:**
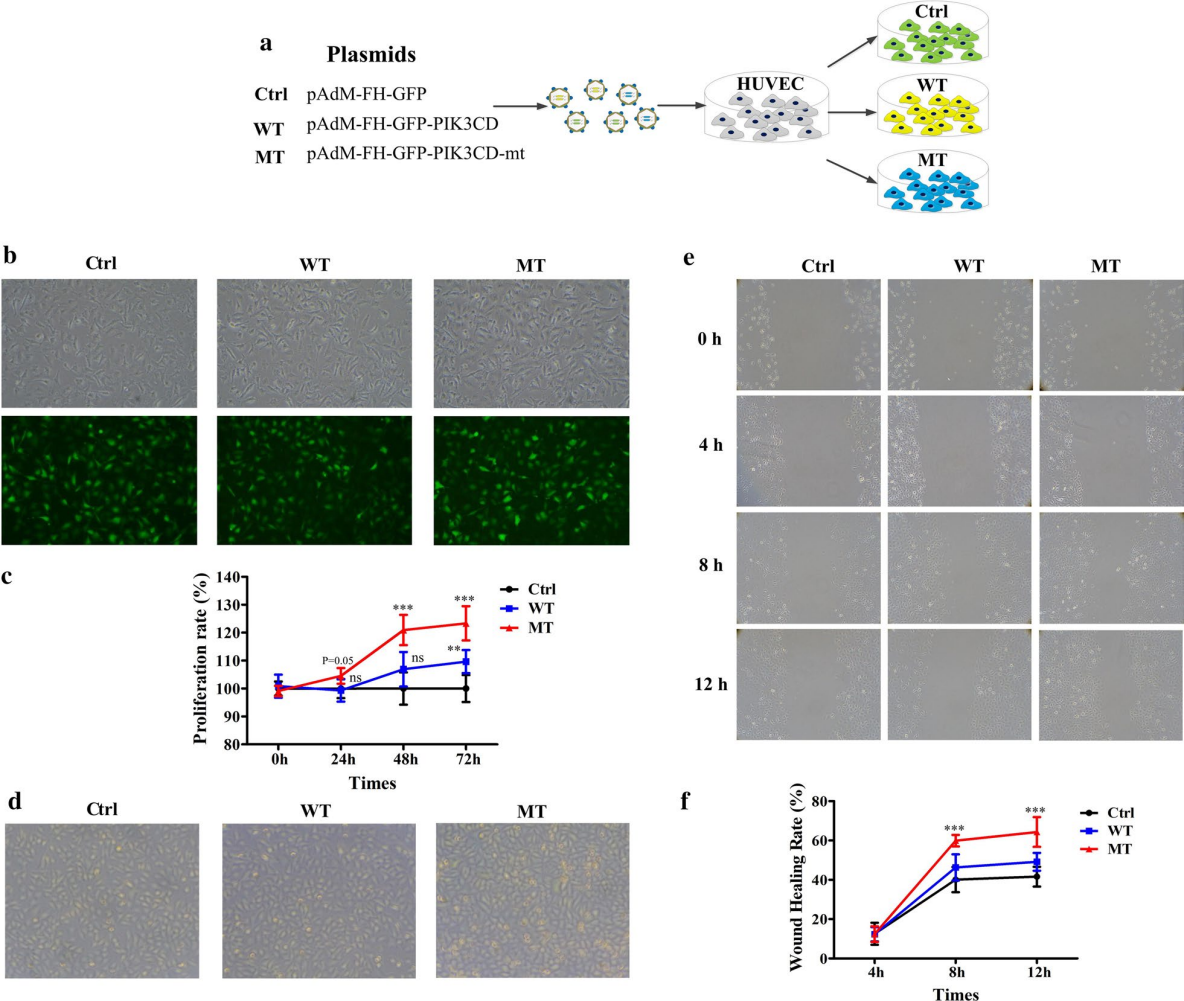
The effects of *PIK3CD* mutation on the proliferation and migration of HUVECs. **a** Schematic diagram showing the construction of HUVECs with stable overexpression of *PIK3CD*. Adenovirus vectors expressing mutant or wild-type *PIK3CD* were constructed. Then, the two overexpression vectors or empty vector was transfected into 293 T cells to produce the virus. The three viruses were infected into HUVECs, resulting in MT-, WT- and Ctrl-HUVECs, respectively, for subsequent experiments. **b** The infection efficiencies of the Ctrl, WT-PIK3CD or MT-PIK3CD vectors was verified by GFP fluorescence imaging. **c**, **d** Cell proliferation of Ctrl-, WT- and MT-HUVECs was evaluated at 0, 24, 48 and 72 h after infection by the CCK8 assay (**c**) and bright-field microscopy (**d**). **e** In the wound healing assay, images of MT-, WT- and Ctrl-HUVECs were acquired at 0, 4, 8 and 12 h after scratching the cell monolayer. **f** The images acquired above were quantitatively analysed by measuring the area of the scratched region lacking cells, and the wound healing rate was calculated as follows: migration area (%) = (A_0_ – A_n_)/A_0_ × 100, where A_0_ represents the area of initial wound area, A_n_ represents the remaining area of wound at the metering time point. Data is representative of three independent experiments and three-five visual fields were randomly selected from the results for quantitative analysis. *p < 0.05, **p < 0.01 and ***p < 0.001 compared with the Ctrl group (Student’s t test)

We performed in vitro functional studies to demonstrate the pathogenicity of the identified *PIK3CD* mutation and how *PIK3CD* affects the movement of endothelial cells. HUVECs overexpressing wild-type and mutant *PIK3CD* (WT- and MT-HUVEC, respectively) via adenovirus infection were created, with empty vector (Ctrl-HUVEC) serving as a control (Fig. 4a). The infection efficiencies were validated as greater than 90% by GFP fluorescence imaging (Fig. 4b). The CCK8 assay was conducted to determine their proliferation capability. The WT-HUVECs presented a similar proliferation rate as the control group, but it appeared to increase significantly at the 72 h time point (Fig. 4c, d). The results of scratch assay showed fastest wound closure in MT-HUVEC (PIK3CD-expressing) compared with vehicle treated (Ctrl) primary HUVEC and wild type at 12 h post scratching. (Fig. 4e, f). These results suggested that exogenous overexpression of mutant *PIK3CD* increased the proliferative and migration capabilities of HUVECs.

### The PIK3CD mutation induced the hyperactivation of the mTOR pathway

**Fig. 5:**
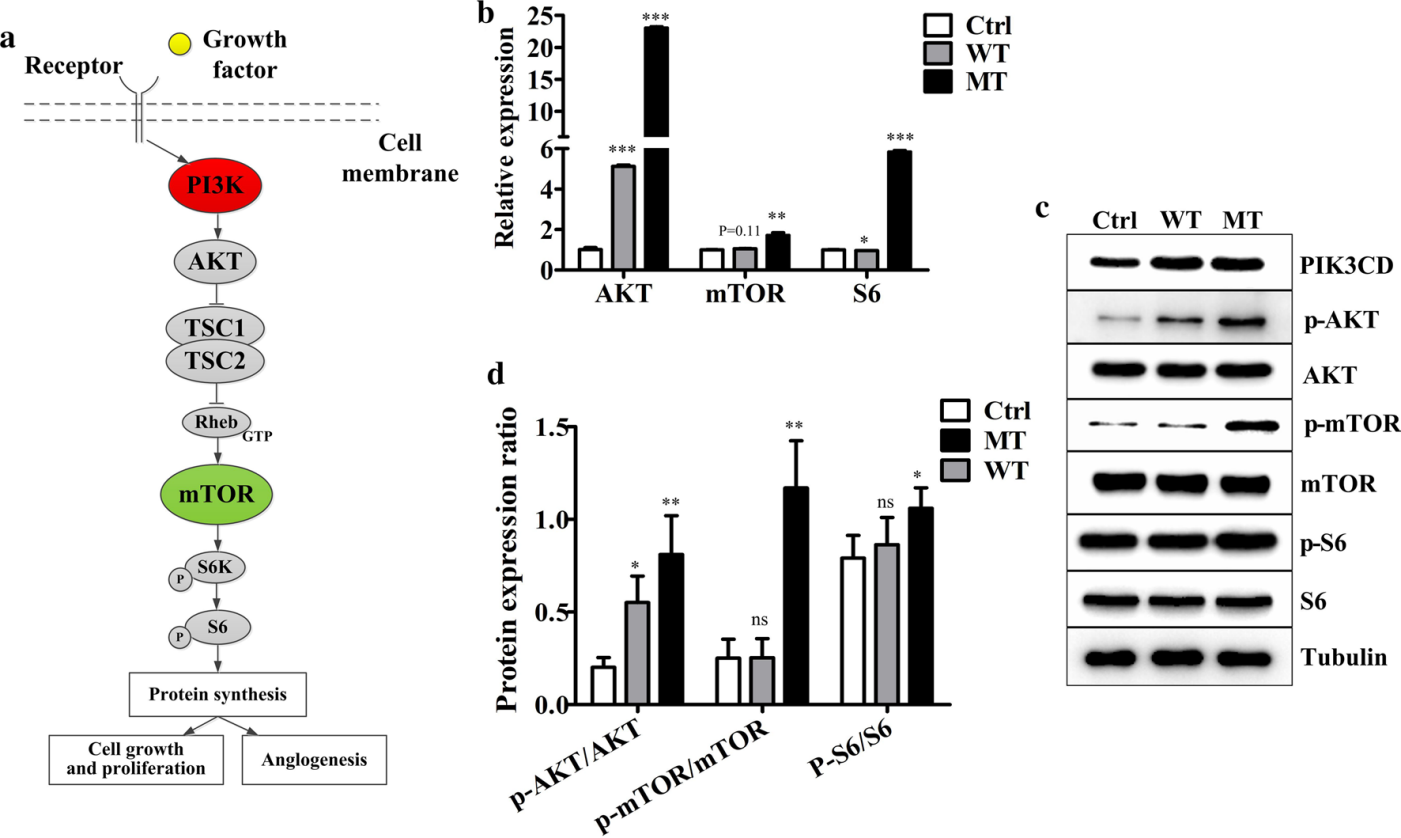
The identified PIK3CD mutation leads to aberrant activation of the mTOR pathway. **a** Schematic figure showing that activation of mTOR is regulated by the upstream proteins PIK3 and AKT. **b** RT-qPCR and **c** immunoblot analysis of AKT, mTOR and S6 mRNA and protein expression, respectively, in Ctrl-, WT- and MT-HUVECs. Ctrl, empty vector; WT, wild-type; MT, *PIK3CD* mutant. **d** Quantification of the protein band intensity. There are no significant differences between WT and Mut group in p-AKT/AKT level (*p* > 0.05). There are significant differences between WT and Mut group in p-mTOR/mTOR (*p* < 0.01). There are no significant differences between WT and Mut group in p-S6/S6 level (*p* > 0.05). Data are presented as the mean ± SD (n = 3–5 per group). *p < 0.05, **p < 0.01 and ***p < 0.001 compared with the Ctrl group (Student’s t test)

We next analysed the activation status of AKT, mTOR and S6, which are critical downstream targets of PI3K (Fig. 5a). Real-time qPCR revealed that the mRNA expression levels of these downstream targets were significantly increased and most pronounced in the MT-HUVECs, followed by the levels in the WT- and Ctrl-HUVECs (Fig. 5b). Furthermore, the protein levels of phosphorylated AKT, mTOR and S6 were strongly upregulated in MT-HUVECs compared with WT- and Ctrl-HUVECs. (Fig. 5c, d). At both the mRNA and protein level, mTOR expression was not affected by the overexpression of wild-type PIK3CD. The results above indicated the significant hyperproliferative phenotypes in MT-HUVECs and the slight increase in proliferation of WT-HUVECs compared with that of the Ctrl-HUVECs. They also supported our hypothesis that this *PIK3CD* mutation is pathogenic.

## Discussion

PI3Ks are a family of lipid kinases with critical roles in cell biology, including cell proliferation, differentiation, migration and survival [15–17]. There are three categories of PI3Ks (Class IA, Class IB; Class II; and Class III). Class IA PI3Ks comprise a p110 catalytic subunit and a p85 regulatory subunit. The p110α, p110β and p110δ catalytic isoforms are encoded by the *PIK3CA*, *PIK3CB* and *PIK3CD* genes, respectively. p110α is frequently involved in human cancers, including endometrial, breast, ovarian, colorectal and other various tumours, by affecting cell proliferation, migration and survival [18–22]. Most of the LMs were caused by somatic mutations in the *PIK3CA* gene, which could lead to the hyperproliferation of lymphatic endothelial cells [7, 8, 13].

Among three Class IA PI3K catalytic isoforms, p110α and p110β are ubiquitously expressed, whereas p110δ is principally enriched in leukocytes and regulates immune functions [23]. However, some non-leucocytes such as neurons [24], ECs (endothelial cells) [25] and lung fibroblasts [26] also express p110δ, albeit at lower levels than in leucocytes. In addition, p110δ is generally overexpressed to induce cancer cell growth and invasion by activating the AKT-mTOR pathway in hepatocellular carcinoma, glioma, glioblastoma, neuroblastoma, colorectal cancer and breast cancer [27–30]. It is well known that LMs present some similar characteristics as tumours, such as uncontrolled cell proliferation and extension into surrounding tissues. Lymphatic vascular endothelial cells in LMs usually exhibit abnormal proliferation due to mTOR activation [31]. However, it was unclear whether genetic changes in *PIK3CD* play a role in the pathogenesis of LMs. All these signs indicated the endothelial cells overproliferated and aggregated, leading to LMs. In the present study, in vitro functional studies demonstrated that exogenous overexpression of wild-type and most significantly mutant *PIK3CD* increased the proliferation rate of HUVECs. In addition, phosphorylated protein levels of AKT, mTOR and S6 were significantly increased in cells with exogenous overexpression of the *PIK3CD* mutant, suggesting that the elevated expression of mutant *PIK3CD* in vascular endothelial cells may promote the overgrowth of endothelial cells and further affect lymphatic vessel development.

To date, LMs are often treated with rapamycin or rapamycin analogues such as everolimus to cure the lesions and improve quality of life [32]. Accepted paper from our laboratory also demonstrated that rapamycin could effectively reduce volume of LMs especially for Macrocystic LMs [33]. Newly paper showed that a combination of VEGFC inhibition with rapamycin is much more potent inducing even LM regression in mice, although this is a contraindication for VEGF inhibition in children [34].

However, given almost 80% LM patients carried *PI3KCA* mutations, mutation-specific inhibitors or combination of inhibitors have become a promising choice for the treatment of LMs. Similar to developmental tumours, LMs carrying a single mutation might be more sensitive to targeted therapies than tumours carrying multiple mutations [35]. Studies have shown that p110α-specific inhibitors could normalize aberrant PI3K signalling, thereby reducing or eliminating PIK3CA-driven vascular malformations. The p110α-specific inhibitor BYL719 was also successfully applied for the treatment of patients with PIK3CA-related overgrowth syndrome, which gives hope to patients with LMs [36]. In the future, we will enrol a larger group of patients with LMs to detect the mutation frequency of *PIK3CD* and to elucidate the mechanism of its pathogenicity. As this is a promising gene for novel targeted therapies, we will also evaluate the effects of *PIK3CD* mutant-specific inhibitors on the reversal of cellular dysfunction.

## Conclusion

In the present study, we identified a novel *PIK3CD* somatic mutation in LM, which could serve as a new candidate pathogenic mutation and is presumably involved in the pathogenesis of LMs. In vitro functional studies demonstrated that exogenous overexpression of mutant *PIK3CD* promoted HUVEC proliferation and migration by activating the mTOR pathway. Therefore, PIK3CD-induced cell proliferation of lymphatic vascular endothelial cells and hyperactivation of mTOR signalling might contribute to the pathogenesis of LMs.

## Materials and methods

### Patients and sample collection

All 6 patients were admitted to and diagnosed by clinicians in the Department of Otolaryngology at Beijing Children's Hospital Affiliated to Capital Medical University. The clinical characteristics were collected from their medical records (Table 1). Guardians of all the participants signed informed consent forms (ICFs) designed in accordance with the Declaration of Helsinki. The tissue specimens of LMs were obtained under the human subject protocol approved by the Human Ethics Committee of Beijing Children's Hospital Affiliated to Capital Medical University (ID: 2019-k-66, approved on February 2019).

### Whole-exome sequencing (WES)

Peripheral blood and tissue specimens of LMs from all 6 children were sent to Running Gene Inc. (Beijing, China) for WES (Additional file 1: Figure S2). Average depth of coverage was × 142 in blood and × 166 in tissue (Additional file 1: Table S1). DNA samples were isolated from the peripheral blood and lymphatic tissue specimens with a DNA Isolation Kit (Bioteke, AU1802 and AU18016). The DNA concentrations were measured with a Qubit dsDNA HS Assay Kit (Invitrogen, Q32851) on a Qubit fluorometer (Invitrogen, Q33216). High-quality DNA samples were fragmented into 200–300 bp by a Covaris Acoustic System (Covaris, Massachusetts, USA), and the resulting DNA fragments were processed with a KAPA Library Preparation Kit (Kapa Biosystems, KR0453) to construct a DNA library. The libraries were estimated with a Qubit dsDNA HS Assay kit (Invitrogen, Q32851), after which hybridization of pooled libraries to the capture probes was conducted with an Agilent SureSelectXT2 Target Enrichment System (Agilent, Santa Clara, USA). Probe-captured DNA fragments were then enriched by post-capture PCR. The final products were sequenced on an Illumina HiSeq X10 platform (Illumina, San Diego, USA) as 150 bp paired-end reads.

Raw data from the HiSeq X10 platform were processed for quality control and then aligned against the human reference genome (GRCh37/hg19) using the Burrows-Wheeler Alignment tool (http://bio-bwa.sourceforge.net/). Duplicate reads were identified using GATK software (www.broadinstitute.org/gatk), and single-nucleotide polymorphisms and insertions and deletions were examined. Low-quality variants were filtered out based on quality by depth (< 2.0), mapping quality (< 40.0), Fisher strand (> 60.0), mapping quality rank sum test (< -12.5) and read position rank sum test (< -8.0). All the called variants were annotated by ANNOVAR (annovar.openbioinformatics.org/en/latest/) based on public databases (1000 Genomes Project, ExAC, gnomAD, ESP6500, CCDS, RefSeq, Ensembl, etc.). The potential impacts of candidate single-nucleotide variants were predicted by the MutationTaster, SIFT, Provean and Polyphen-2 programs.

### Germline mutations involved in either PI3K/AKT/mTOR or Ras pathways

Low-quality variants were filtered out based on quality by depth (< 8.0). The remaining variants were filtered against 1000 Genomes Project_EAS, ExAC and gnomAD, with a minor allele frequency (MAF) < 1% for autosomal and X-linked recessive mutations and an MAF < 0.01% for autosomal and X-linked dominant mutations. Based on the Human Gene Mutation Database, nonsense, frameshift, and splicing mutations annotated as disease mutations were retained. Only candidate genes associated with both PI3K/AKT/mTOR and Ras pathways were included. No definite pathogenic germline variant was identified.

### Somatic mutations involved in either PI3K/AKT/mTOR or Ras pathways

Germline mutations appearing in the peripheral blood were filtered out. The remaining mutations were selected based on quality by depth (< 8.0) and against 1000 Genomes Project_EAS, ExAC and gnomAD, with an MAF < 0.01%. Only exonic and splicing variants were included. Synonymous variants and variants with low number of alteration (alt < 4) were excluded as well. Finally, only candidate genes associated with the both PI3K/AKT/mTOR and Ras pathways were included.

### Digital polymerase chain reaction (PCR)

Digital PCR were conducted to verify the remaining *IRS1*, MTOR, *TSC1*, TSC2, *PIK3CA* and *PIK3CD* variants. DNA samples were mixed with 2X ddPCR Supermix for probes (Bio-Rad Laboratories, Inc., USA), probes, primers and ddH2O (Table 3). The mixture and droplet generation oil (Bio-Rad Laboratories, Inc., USA) were separately loaded on the DG8 cartridge. Then, targeted droplets were generated by a QX200 Droplet Digital PCR system (Bio-Rad Laboratories, Inc., USA) and transferred to 96-well plates. After PCR in a Bio-Rad thermal cycler T100, the digital PCR data were read and briefly analysed on the QX200 Droplet Digital PCR system.

**Table 3:** Sequence of primers and probes used for digital polymerase chain reaction (ddPCR)

Primer and probes	Sequence (5′-3′)
*PIK3CA-F*	GCTCAAAGCAATTTCTACACGA
*PIK3CA-R*	CTTACCTGTGACTCCATAGAAAATC
*PIK3CA-P-G*	6-FAM- TGAAATCACTGAGCAGGA- BHQ-X
*PIK3CA-P-A*	HEX- TGAAATCACTAAGCAGG- BHQ-X
*PIK3CD-F*	TCCGAGATGCACGTGCC
*PIK3CD-R*	CCTTCATGTGGTGGGTGCT
*PIK3CD-P–T*	6-FAM -TTCGGCCTCATCCT- BHQ-X
*PIK3CD-P–C*	HEX -TTCGGCCCCATCC- BHQ-X

### Cell culture and infection

Human umbilical vein endothelial cells (HUVECs) were donated by Beijing Belife Bio-Medical Technology LTD and cultured in endothelial cell medium (ECM) (cat no. 1001; ScienCell, San Diego, California, USA) supplemented with 5% foetal bovine serum (FBS) (cat no. 0025; ScienCell, San Diego, California, USA), 1% Endothelial Cell Growth Supplement (ECGS) (cat no. 1052; ScienCell, San Diego, California, USA) and 1% penicillin/streptomycin solution (cat no. 0503; ScienCell, San Diego, California, USA). Cells were cultured at 37 °C in a humidified atmosphere containing 5% CO_2_.

cDNAs coding wild-type *PIK3CD* (GenBank accession NM_005026.4) or *PIK3CD* mutations were synthesized for adenovirus packaging (Vigene Bioscience, Shandong, China). The vector used was a bi-cistronic construct with EGFP. HUVECs were seeded in each well of 6-well plate (5 × 10^3^ cells/well). Approximately 18–24 h later, the medium was replaced with fresh medium containing different viruses (final concentration, 2.5 × 10^6^ pfu/mL). At 48 h after initial virus treatment, the infection efficiency was evaluated using GFP fluorescence imaging (Additional file 1: Figure S3).

### Cell viability assay

Infected cells (5 × 10^3^/well) in 100 μL of culture medium were seeded into 96-well plates and incubated for 18–24 h, after which the medium was replaced with a virus suspension (final concentration 2.5 × 10^6^ pfu/mL) in fresh medium. Afterward, the cell viability assay was performed by adding 10 µL of reagent from Cell Counting Kit-8 (CCK8) (Meilunbio, cat no. MA0218-L, Dalian, China) into each well and incubating the plates for another 2 h; then, absorbance at 450 nm was detected with a microplate reader (Molecular Devices, Silicon Valley, CA, USA). The cell survival rate was calculated as follows: (OD value of wild-type PIK3CD or mutant PIK3CD group/OD value of the control group) × 100%.

### Quantitative real-time PCR (RT-qPCR)

Total RNA was isolated from HUVECs using an RNA isolation kit according to the manufacturer's protocol (cat no. 220010; Shanghai feijie biological, Inc, Shanghai, China). The RNA was subsequently reverse transcribed into cDNA using a KR106-02 reverse transcription system according to the manufacturer’s instruction (cat. no. KR106-02; TIANGEN, Beijing, China). To detect RNA expression, qPCR analyses were carried out in triplicate using SYBR Green PCR Master Mix (cat. no. FP205-02; TIANGEN, Beijing, China) and run on a Roche LightCycler 96 (Roche Diagnostics, Indianapolis, IN, USA). Relative expression was calculated by the 2 − ΔΔCt method with GAPDH as the endogenous control. The primer sequences for the specific targets were showed in Table 4.

**Table 4:** Sequence of primers used for quantitative real-time PCR *(RT-qPCR)*

Primer	Sequence (5′-3′)
*GAPDH-F*	5ʹ- GGAGCGAGATCCCTCCAAAAT -3ʹ
*GAPDH-R*	5ʹ- GGCTGTTGT CATACTTCTCATGG -3ʹ
*mTOR-F*	5ʹ- ATGCTTGGAACCGGACCTG -3ʹ
*mTOR-R*	5ʹ-TCTTGACTCATCT CTCGGAGTT -3ʹ (reverse)
*AKT(Human)-F*	5′-CTACCCACACAGCAGTACGC-3'
*AKT(Human)-R*	5′-AAGTCGCTGGTGTTAAGCCG-3'
*S6-F*	5ʹ- AGGGTTATGTGGTCCGAATCA -3ʹ
*S6-R*	5ʹ- TTGGTCTGT AACAGGAATGCC -3ʹ

### Western blot

Total proteins were extracted from HUVECs using RIPA buffer (cat. no, XSY-WB-001; B-Belife, Beijing China) mixed with 1% protease inhibitor cocktail (cat. no. 04693116001; Roche Molecular Biochemicals, Mannheim, Germany). The protein concentration was determined using a BCA Protein assay kit (cat. no. CW0014S; CWBIO, Beijing, China). Protein extracts were then mixed with 5 × SDS loading buffer and boiled for 10 min. Then 20–50 μg per sample were separated via SDS-PAGE under reducing or non-reducing conditions on a 10% polyacrylamide gel and then electrotransferred onto PVDF membranes (Millipore, Bedford, MA, USA) in vertical buffer tanks. The membranes were blocked with 5% non-fat milk in TBST buffer (50 mM Tris–HCl (pH 7.4), 0.9% NaCl, and 0.1% Tween 20) before they were incubated with primary antibodies (Table 5) for 2–3 h at room temperature or overnight at 4 °C. After the addition of the HRP-conjugated secondary antibodies for 1 h, signals were detected with an electrogenerated chemiluminescence (ECL) detection reagent (cat. no. MA0186; Meilunbio, Dalian, China). Relative target protein expression levels were normalized to those of tubulin and visualized using ImageJ software.

**Table 5:** Antibodies for western blot

No	Antibodies	Company	No. product
1	Anti-p-AKT antibody	Abcam	ab8933
2	Anti-AKT antibody	Abcam	ab8805
3	Anti-p-mTOR antibody	Abcam	ab109268
4	Anti-mTOR antibody	Abcam	ab32028
5	Anti-p-S6 antibody	CST	4858
6	Anti-S6 antibody	CST	2217

### Wound healing assay

The migration ability of HUVECs were assessed by the wound healing assay. A sterile tip was used to create a wound in a cell monolayer, which was then washed 3 times to remove non-adherent cells, and fresh medium was added to the cultures. Images were captured at 0, 4, 8 and 12 h after scratching. Photoshop software (Adobe Photoshop CS6) was used to measure the area of the wound at each time point and calculate the wound healing rate as follows: migration area (%) = (A_0_ − A_n_)/A_0_ × 100, where A_0_ represents the area of initial wound area, an represents the remaining area of wound at the metering time point.

### Statistical analysis

All in vitro experiments performed in this study were repeated three times. Statistical analysis was performed using GraphPad Prism software, and all comparisons between groups were assessed using Student’s t test. All clinical data are indicated as mean ± standard deviation (Mean ± SD), with the significant statistical threshold of two-tailed *p* value < 0.05.

## Supplementary Information

**Additional file 1: Figure S1**. Pathological structure of each subtype of LM.** Figure S2**. Schematic diagram of the bioinformatics pipeline to analyze the targeted deep sequencing data.** Figure S3**. Transfection rate quantified by qPCR.** Table S1**: The coverage of each sample and average depth.

## Data Availability

The datasets used and analysed during the current study are available from the corresponding author on reasonable request.
