# Correction to: A somatic mutation in *PIK3CD* unravels a novel candidate gene for lymphatic malformation

**DOI:** 10.1186/s13023-021-01946-7

**Published:** 2021-07-19

**Authors:** Shengcai Wang, Wei Wang, Xuexi Zhang, Jingang Gui, Jie Zhang, Yongli Guo, Yuanhu Liu, Lin Han, Qiaoyin Liu, Yanzhen Li, Nian Sun, Zhiyong Liu, Jiangnan Du, Jun Tai, Xin Ni

**Affiliations:** 1 Department of Otolaryngology-Head and Neck Surgery, National Center for Children’s Health, Beijing Children’s Hospital, Capital Medical University, Beijing, 100045 China; 2 Laboratory of Tumor Immunology, National Center for Children’s Health, Beijing Pediatric Research Institute, Beijing Children’s Hospital, Capital Medical University, Beijing, 100045 China; 3 Beijing Key Laboratory for Pediatric Diseases of Otolaryngology, Head and Neck Surgery, National Center for Children’s Health, Beijing Pediatric Research Institute, Beijing Children’s Hospital, Capital Medical University, Beijing, 100045 China; 4 Running-Gene Inc, Health Valley 602, Beijing, China

## Correction to: *Orphanet Journal of Rare Diseases* (2021) 16:208 10.1186/s13023-021-01782-9

Following the publication of the original article [1] it was brought to our attention that the joint first authorship and equal contribution of Shengcai Wang, Wei Wang and Xuexi Zhang, was not indicated in the published article.

The joint first authorship and equal contribution of the three co-authors has now been marked with dagger symbols in the author list and an explanatory footnote at the end of this Correction.

The original article has also been corrected as above.
