# Supplementary material for: A somatic mutation in PIK3CD unravels a novel candidate gene for lymphatic malformation
Source: Orphanet J Rare Dis. 2021 May 8;16:208. doi: 10.1186/s13023-021-01782-9 (PMC8106842; doi:10.1186/s13023-021-01782-9)
Supplement: Supplementary file 1 — Additional file 1: Figure S1. Pathological structure of each subtype of LM. Figure S2. Schematic diagram of the bioinformatics pipeline to analyze the targeted deep sequencing data. Figure S3. Transfection rate quantified by qPCR. Table S1: The coverage of each sample and average depth. [file 13023_2021_1782_MOESM1_ESM.docx]

**Supplementary Materials:** Figure S1: Pathological structure of each subtype of LM; Figure S2: Schematic diagram of the bioinformatics pipeline to analyze the targeted deep sequencing data. Figure S3: Transfection rate quantified by qPCR and quantification of GFP. Table S1 The coverage of each sample and average depth


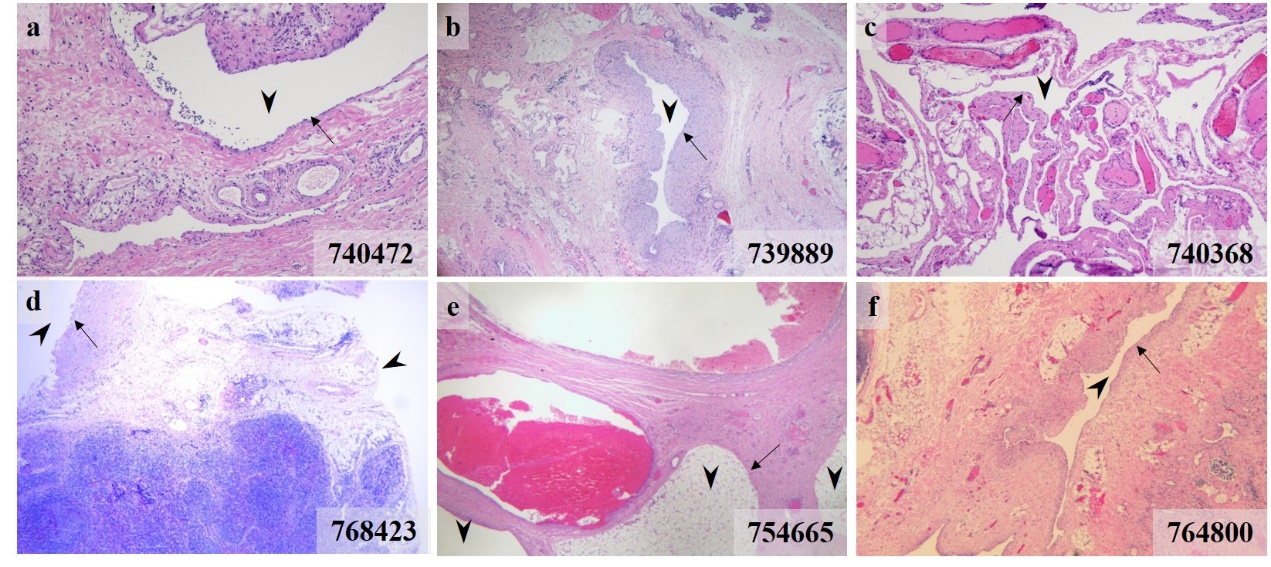


Figure S1. Pathological structure of each subtype of LM. (a, b) Macrocystic LMs comprise thin-walled lymphatic vessels with ectopic smooth muscle cells, are irregularly shaped, and vary in size. (c, d) Microcystic LMs are composed of vascular spaces with a single layer of flattened endothelium lining the walls of the lymphatic channels. The fibrovascular vessels are different in size, with few smooth muscle cells. (e, f) Mixed cystic LMs contain the same lymphatic endothelial cells lining the lymphatic walls. The fine arrow shows the lymphatic endothelium, and arrowhead shows the lymphatic lumen. Haematoxylin and eosin; magnification 10X.


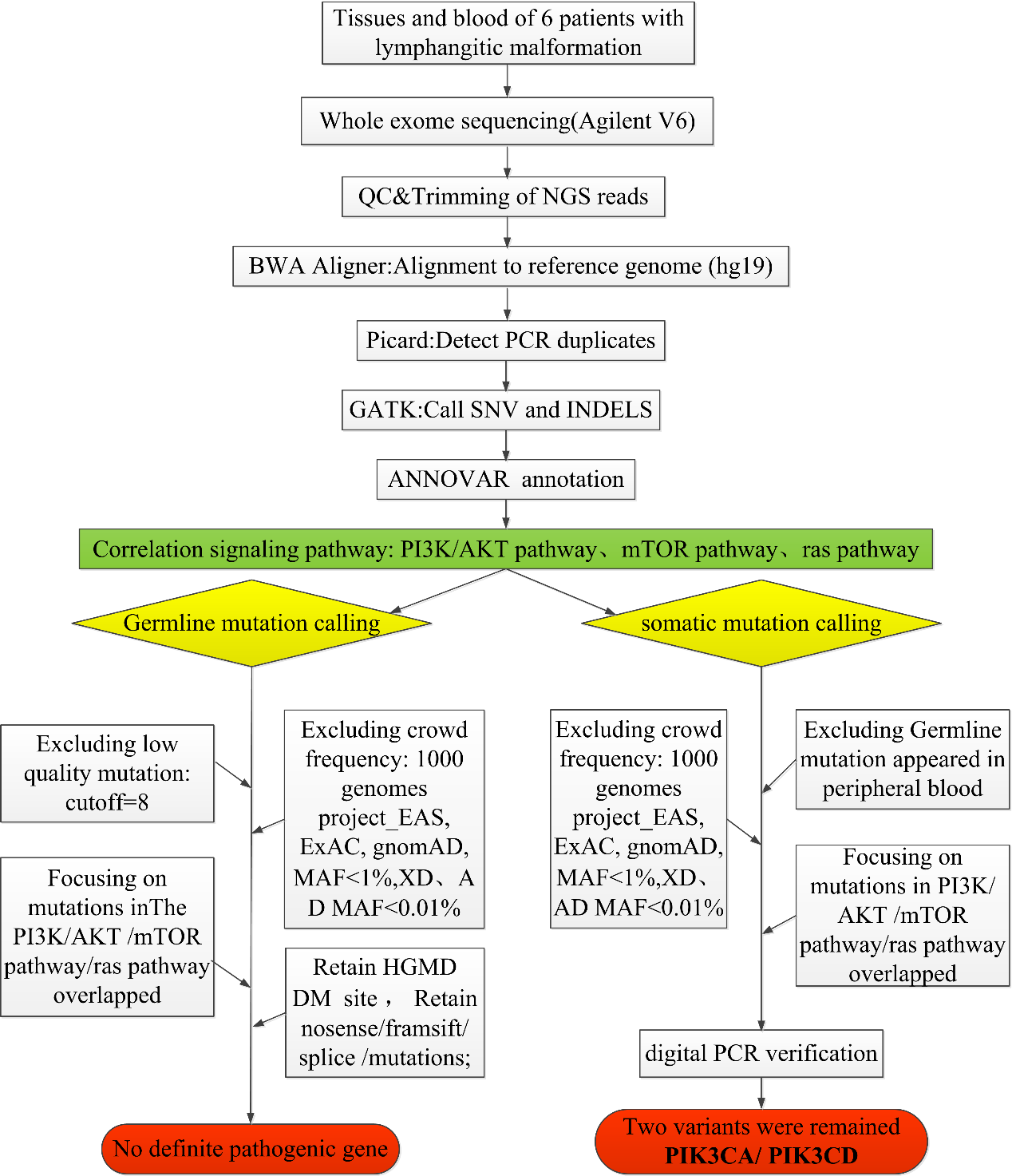


Figure S2. Schematic diagram of the bioinformatics pipeline to analyze the targeted deep sequencing data.


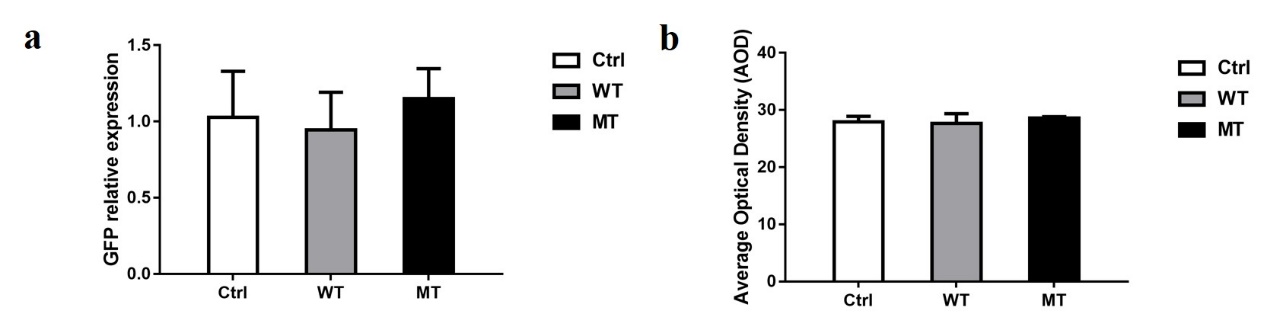


Figure S3: Transfection rate quantified by qPCR (a) and quantification of GFP Fluorescence photos of the transfected cells are taken, and then quantified the different visual fields of the photos with Image J software (b). It was found that there was no significant difference between the groups. The expression of GFP mRNA was detected by Q-PCR. There was no significant difference among the groups. Therefore, we think that the infection efficiency of each group is close, so we can compare the infection efficiency of each group.

Table S1 The coverage of each sample and average depth

| **Patient** | **Coverage** | **Average sequencing depth** | **Fraction of target covered with at least 20X** | **Total sites** | **Exclude control (Somatic)** | **Depth>=8, MAF<0.001, include exonic and splicing variant, exclude synonymous, exclude low number of alt (alt<4)** | **mTOR-associated** |
| --- | --- | --- | --- | --- | --- | --- | --- |
| 740472  blood | 100.00% | 192.31 | 99.40% | 350521 | 90195 | 13 | 2 |
| 740472  tissue | 100.00% | 147.47 | 98.80% | 239615 |  |  |  |
| 740368  blood | 100.00% | 217.18 | 99.50% | 286952 | 132955 | 25 | 6 |
| 740368  tissue | 100.00% | 188.63 | 99.50% | 304841 |  |  |  |
| 739889  blood | 99.90% | 112.43 | 97.40% | 409722 | 360038 | 29 | 2 |
| 739889  tissue | 100.00% | 232.15 | 99.10% | 618931 |  |  |  |
| 754665  blood | 99.70% | 159.93 | 98.70% | 454392 | 268987 | 25 | 3 |
| 754665  tissue | 99.70% | 179.59 | 98.80% | 545856 |  |  |  |
| 768423  blood | 99.90% | 80.33 | 95.70% | 253686 | 459477 | 13 | 1 |
| 768423  tissue | 99.90% | 108.64 | 96.70% | 639567 |  |  |  |
| 764800  blood | 99.90% | 90.46 | 96.70% | 526084 | 114732 | 23 | 2 |
| 764800  tissue | 99.90% | 137.15 | 94.70% | 304581 |  |  |  |

More than 2 variants were found but the rest of them were filtered out. A total of 16 variants associated with mTOR pathway were identified in 6 patients. Nineteen variants were located in 6 genes, including IRS1, MTOR, PIK3CA, PIK3CD, TSC1 and TSC2. All of them were validated by ddPCR but only 2 variants in PIK3CA and PIK3CD were positive.
